# Ammonia Valorization by Liquid–Liquid Membrane Contactors for Liquid Fertilizers Production: Experimental Conditions Evaluation

**DOI:** 10.3390/membranes12070663

**Published:** 2022-06-27

**Authors:** Mònica Reig, Xanel Vecino, Miguel Aguilar-Moreno, César Valderrama, José Luis Cortina

**Affiliations:** 1 Chemical Engineering Department, Escola d’Enginyeria de Barcelona Est (EEBE), Campus Diagonal-Besòs, Universitat Politècnica de Catalunya (UPC)-BarcelonaTECH, C/Eduard Maristany 10-14, 08930 Barcelona, Spain; xanel.vecino@uvigo.es (X.V.); miguel.aguilar.moreno@upc.edu (M.A.-M.); cesar.alberto.valderrama@upc.edu (C.V.); jose.luis.cortina@upc.edu (J.L.C.); 2 Barcelona Research Center for Multiscale Science and Engineering, Campus Diagonal-Besòs, 08930 Barcelona, Spain; 3 Research Center in Technologies, Energy and Industrial Processes (CINTECX), Chemical Engineering Department, Campus As Lagoas-Marcosende, University of Vigo, 36310 Vigo, Spain; 4 Water Technology Centre (CETaqua), Carretera d’Esplugues, 75, 08940 Cornellà de Llobregat, Spain

**Keywords:** acid stripping, concentration, volume, temperature, ammonium salts, recovery

## Abstract

Liquid–liquid membrane contactors (LLMCs) were studied as a sustainable technology for ammonia recovery from wastewater. Ammonia can be valorized by LLMCs as a potential nutrient and produce liquid fertilizers. Thus, this work aims for the study of different experimental LLMC conditions to produce ammonium salts by an acid stripping stream. The experiments were conducted using two 3M^TM^Liqui-Cell^TM^ LLMC in a series, located in the vertical position and using HNO_3_ as the acid stripping solution. The flow rates for the feed and stripping sides were fixed during the tests, and two steps were conducted based on previous works. However, different experimental conditions were evaluated to determine its effect on the overall performance: (i) replacing the feed or stripping solution between the steps, (ii) the initial ammonia concentration of the feed solution, (iii) feed volume and (iv) feed temperature. The results demonstrated that better achievements were obtained replacing the acid stripping solution between steps, whereas the feed temperature did not substantially affect the overall performance. Additionally, a high initial ammonia concentration provided more ammonia recovery, although the concentration factor achieved was higher for the low initial ammonia concentration. Finally, a high feed volume afforded better results for the fertilizer side, whereas more NH_3_ recovery was achieved using less feed volume.

## 1. Introduction

Liquid–liquid membrane contactor (LLMC) is a promising and sustainable technology for nitrogen recovery from water or wastewater resources in comparison with other processes such as adsorption or biological oxidation, among others, where recovery is more difficult or is not possible [1]. Regarding LLMCs, the use of hollow fibers (HF), which are usually hydrophobic and microporous, is common [2,3], although there are some studies that used tubular membranes for treating fouled systems [4,5]. Moreover, due to the hydrophobic feature of HF membranes, polytetrafluoroethylene (PTFE) and polypropylene (PP) materials are usually used, and more recently, polyvinylidene fluoride (PVDF) has been proposed. The former materials are symmetric membranes, and the latter are asymmetric, which provides less mass transfer resistance [2,6]. In fact, a higher interfacial area per unit volume or the control of the flow rates are advantages for the use of hydrophobic HF-LLMCs in comparison with conventional processes (such as absorption) [2].

It is worth mentioning that, to recover nitrogen from wastewater streams by using HF-LLMCs, these streams should be at a pH above 9.3 (pKa) to assure that nitrogen is an ammonia gas form [3]. In fact, the driven force of HF-LLMCs is the chemical reaction of an acid stripping solution with ammonia gas due to the concentration or vapor pressure differences between the two sides of the membrane [1,3]. Indeed, the ammonia gas passes through the hydrophobic membrane from the feed (named the shell side) to the acid stripping solution (called the lumen side) by diffusion phenomena. Thus, it is possible to transform the ammonia present in these resources into ammonium salts, which could be used as liquid fertilizers [1,3]. Sulfuric acid, nitric acid and phosphoric acid are acid stripping solutions used in HF-LLMC processes, the first acid being the most frequently applied [3,7]. However, the bottleneck of the HF-LLMC processes is the control of the membrane wettability; if the liquid pressure exceeds the breakthrough pressure, the pores of the hydrophobic membrane get wet [1]. For that reason, there have been several studies where different operational parameters, configurations, membrane types, etc. were tested to maximize the ammonia recovery and minimize the water passage. For example, Zhu et al. [8] evaluated the effect of pH and the viscosity of the feed solution (containing 2 g NH_3_/L) on the mass transfer in two different PP HF-LLMCs. It was concluded that viscosity is not the main factor affecting the rate of mass transfer, while the pH of the feed had a significant effect on the rate of mass transfer, the removal efficiency, and the flux of ammonia. The authors suggested that the highest treating efficiency was achieved when the initial pH value of the feed solution was adjusted over 11. Licon et al. [9] studied the influence of various operational parameters (i.e., flow rate, initial ammonia concentration and stripping acid concentration) for ammonia recovery from tertiary effluents by zeolites that generate basic ammonia concentrates (up to 1–3 g NH_3_/L in 1–2 g NaOH/L) by using PP HF-LLMC. It was concluded that the ammonia mass transfer did not vary substantially as a function of the initial ammonia concentration (0.3–1.7 g/L) and flow rate (7.59–11.06 cm^3^/s), and the reaction was only affected by the excess strong acid (nitric or phosphoric) used.

Reig et al. [10] tested different parameters using PP HF-LLMC for ammonia valorization as follows: position (horizontal and vertical), feed and acid streams inputs (shell and lumen), type of acid stripping solution (H_3_PO_4_ and HNO_3_), membrane drying, the flow rate for each stream (263–770 mL/min), number of steps (1 and 2) and number of membrane contactors (1 and 2 in a series). The treated urban wastewater stream contained high contents of ammonia (around 4.5 g NH_3_/L). The authors selected a one-step configuration using two vertical membrane contactors in a series, using the shell side for the feed stream and the lumen side for the acid stripping solution (HNO_3_) at 450 mL/min and 770 mL/min flow rates for the feed and acid stripping solutions, respectively, to obtain the maximum ammonia recovery (>95%). In the abovementioned works, the most commonly studied HF-LLMCs were those provided by 3M Company (Saint Paul, MN, USA) under the tradename Liqui-Cell, which are made with PP membranes. However, recently, Sheikh et al. [11] tested two novel HF-LLMCs modules containing S-type (named A60 with a skin layer with low porosity) and Q-type (called Q-A60 with a skin layer with high porosity) fibers supplied by Separel DIC Corporation (Tokyo, Japan). Both types of fibers, with asymmetric, porous, and hydrophobic membranes made from poly(4-methyl-1-pentene) (PMP), were used as an efficient technology for ammonia recovery by producing liquid fertilizers. The results showed that in terms of the N% recovered, the performance of the Q-type PMP-HF-LLMC module was better than the S-type, since it obtained a higher ammonia mass transfer in a shorter time. This could be because the Q-type module has a skin layer with higher porosity than the S-type.

Therefore, in view of the above, it is still interesting to study different operational parameters, such as (i) replacing the feed or the stripping solution between the steps, (ii) the initial ammonia concentration of the feed solution (4.5 and 1.0 g/L), (iii) feed volume (60 and 5 L) and (iv) feed temperature (25 and 35 °C) to evaluate the effects of them on the PP HF-LLMC efficiency of ammonium recovery from wastewater streams. In fact, to the best of our knowledge, some of these parameters have scarcely been previously studied, such as the feed volume effect on the PP HF-LLMC performance.

## 2. Materials and Methods

### 2.1. Reagents

Nitric acid (65%, HNO_3_) was used as the acid stripping solution for ammonium salts production. Additionally, methanesulfonic acid (CH_3_SO_3_H, 99%), sodium hydrogen carbonate (NaHCO_3_, 99%) and anhydrous sodium carbonate (Na_2_CO_3_, 99%) were used for the ionic chromatography analysis. All chemicals used in this work were analytical grade reagents and were supplied by Sigma-Aldrich (Madrid, Spain).

#### Wastewater Solution

Pretreated urban wastewater from Vilanova i la Geltrú’s wastewater treatment plant (WWTP) was used in this work (Barcelona, Spain). In this case, the produced wastewater was finally treated in a pilot plant, located in the same installation, to reduce the ammonium levels by zeolites [7,10,12]. However, the main drawback of this final treatment was the production of a more concentrated stream, which was rich in ammonia due to the high pH (≈12). Thus, this pretreated stream was used as the feed solution for this work.

### 2.2. Experimental Set-Up

Although the experimental set-up has already been described elsewhere [7,10,13], the most relevant details about the lab-scale LLMC experimental set-up are described in this section. A pair of 2.5 × 8 Liqui-Cel^®^ Membrane Contactor X-50 polypropylene (3M^TM^, USA) LLMC modules were used located in a series and the vertical position. The abovementioned modules and their characteristics (e.g., membrane configuration, active area, hydrophobicity, pore diameter or the number of fibers) have also been described previously [9], but it is worth mentioning that the configuration was hollow fibers with a membrane area of 1.4 m^2^. Two tanks were used to introduce the feed solution (60 L) and the acid stripping solution (0.5 L), connected to the LLMC modules through PVC flexible tubes. All tests were conducted under contra-current mode in a closed-loop (i.e., recirculating both streams and introducing the feed solution through the shell side and the acid stripping one through the lumen (inside the fibers). Furthermore, it should be noted that both flow rates were kept constant following the optimal results previously determined: 450 mL/min for the feed stream and 700 mL/min for the acid stripping side.

As a summary, the hydrophobic LLMCs used in this work only allowed the passage of ammonia gas (pH > pKa(NH_4_^+^/NH_3_) = 9.3) from the feed solution through the hollow fibers. Then, the NH_3_ reacts with the acid stripping solution, producing ammonium salts, which can be used in agriculture as liquid fertilizers. In this case, HNO_3_ was used as an acid stripping solution, keeping the produced solution between pH 2 and 3 [9] by a concentrated HNO_3_ (65%) addition. For that, 0.5 L of 0.4 M HNO_3_ was prepared as the initial acid stripping solution, which, after the trials, was converted into ammonium nitrate salts, as follows in Equation (1).

(1) $${\mathrm{NH}}_{3\left(g\right)}+{\mathrm{HNO}}_{3}↔{\mathrm{NH}}_{4}{\mathrm{NO}}_{3}$$

Additionally, in order to improve the ammonia recovery and the fertilizer concentrations, the valorization process was carried out in 2 steps, i.e., once the feed concentration reached a plateau (meaning that no more ammonia could be transported to the stripping side), the experiment was interrupted, and the acid or the feed solution was changed for a new one. In fact, based on the previous work [10], the only solution that changed between steps was acid stripping, to be able to decrease the ammonia concentration of the feed solution even more, although the ammonium salt concentration did not increase. However, in this work, the change of the feed solution was also considered a variable of study to be able to increase the fertilizer concentrations and determine which of both options would lead to the optimal overall performance.

Furthermore, not only the replacing of the acid stripping or the feed solution between both stages was studied to improve the ammonia valorization as ammonium salts but also other variables, such as the initial ammonia concentration in the feed solution (side or mainstream, around 4.5 or 1 g NH_3_/L, respectively), feed volume to be treated (60 or 5 L) and feed temperature (around 25 °C (room) or 35 °C (maximum allowed by the 3M^TM^ LLMC)).

During the experiments, several samples were collected from both tanks over time. Then, these samples were analyzed to determine their compositions (mainly the concentration of ammonia in the feed solution and the concentration of ammonium and nitrate in the acid stripping solution). All experiments were carried out in duplicate. Thus, data were reported as the mean ± standard deviation of replicate determinations.

#### Experimental Design

Five experiments were designed to study the effects of the abovementioned parameters. Table 1 summarizes the experimental design, where one parameter was varied trial after trial.

As can be seen in Table 1, the first experiments (Exp. 1 and 2) were designed to study the effect of changing the acid or feed solution after the plateau stage. In this case, 60 L of sidestream wastewater at room temperature were used (around 4.5 g NH_3_/L) following the already published conditions [7,10]. Once the best option was decided, the next experiment (Exp. 3) was designed to study the effect of the initial ammonia concentration by using mainstream wastewater (around 1 g NH_3_/L), keeping the other parameters as in the first experiments. Next, the feed volume was varied from 60 to 5 L (Exp. 4) to determine its effect on the overall performance. Finally, the feed temperature was studied by increasing the feed solution temperature up to 35 °C (Exp. 5).

### 2.3. Data Analysis

Four main parameters were determined to analyze the LLMC efficiency, depending on the analyzed parameters, to valorize ammonia from wastewater and recover it as liquid fertilizers: two for the feed side and two more for the acid stripping side.

Thus, the final ammonia concentration and the ammonia recovery were the analyzed parameters for the feed side. The former was directly analyzed by analytical methodologies, and the latter was calculated by Equation (2) [7]:

(2) $$\mathrm{Ammonia}\;\mathrm{recovery}\;\left(\%\right)=\frac{C_{\mathrm{feed},0}-C_{\mathrm{feed},\mathrm{final}}}{C_{\mathrm{feed},0}}\cdot 100$$

where C_feed,0_ and C_feed,final_ are the initial and final ammonia concentrations (mg/L), respectively, in the feed tank.

On the other hand, the ammonia concentration factor (CF) and the final nitrogen concentration in the acid stripping side were also determined. Indeed, CF considered the initial concentration of ammonia in the feed solution and the obtained concentration of ammonia in the liquid fertilizers. Thus, the CF was calculated following Equation (3) [7]:

(3) $$\mathrm{CF}\;\left(-\right)=\frac{C_{f\left({\mathrm{NH}}_{3},\mathrm{acid}\;\mathrm{tank}\right)}}{C_{0\left({\mathrm{NH}}_{3},\mathrm{feed}\;\mathrm{tank}\right)}}$$

where C_0(NH3,feed tank)_ and C_f(NH3,acid tank)_ are the initial and final NH_3_ concentrations (mg/L) in the feed and acid stripping tanks, respectively.

Finally, the ammonium salt composition was expressed by the percentage of N-NH_4_ present in the liquid fertilizer solution, as described by Equation (4) [7]:

N-NH_4_ concentration (%, *w*/*w*) = C_acid stripping,final_ (4)

where C_acid stripping,final_ is the final ammonium concentration in the acid stripping tank (g N-NH_4_/g solution (*w*/*w*)).

The %N in the liquid fertilizer is a common parameter that fertilizer companies consider when describing the composition of their liquid fertilizers (e.g., Fertiberia, https://www.fertiberia.com/, accessed on 2 May 2022), instead of ammonium salt amount or concentration. Thus, this parameter was used to determine the composition of the obtained ammonium salts (fertilizer).

### 2.4. Analytical Methodology

During the experiments, the pH was monitored and measured online by a GLP 22 pH meter (Crison, Alella, Spain), and the conductivity was measured by an EC-Metro GLP 31 (Crison) [7]. The total carbon (TC) was determined by a TOC-V_CPH_ meter (Shimadzu, Kyoto, Japan).

Moreover, the sample compositions were determined by ionic chromatography. In this case, two apparatuses from Thermo-Fisher Scientific (Waltham, MA, USA) were used for cation and anion quantifications: (i) Dionex ICS-1000 equipped with a CS16 column (5 × 250 mm), a pre-column CG16 (5 × 50 mm) and cationic detector ICS-1000 and (ii) Dionex ICS-1100 equipped with a AS23 column (4 × 250 mm), pre-column AG23 (4 × 50 mm) and an anionic detector ICS-1100. Thus, 0.03 mol/L of the CH_3_SO_3_H solution was used as the mobile phase for the cations equipment and a mixture of 0.8 mmol/L of NaHCO_3_ and 4.5 mmol/L of Na_2_CO_3_ for the anions system. Both devices were controlled by Chromeleon^®^ chromatographic software.

## 3. Results and Discussion

First of all, pretreated wastewater from the WWTP was analyzed by ionic chromatography to determine the ions concentration and other parameters, such as pH or conductivity (Table 2).

As can be seen in Table 2, the sidestream wastewater used as the feed solution in this work was mainly composed of sodium and ammonium ions mixed with dissolved organic matter at a high pH (>pKa = 9.3). Then, ammonium was present as ammonia in gas form. Moreover, other elements were found, such as potassium, chloride, nitrate or sulphate, but at trace levels. On the other hand, apart from the sidestream water, mainstream wastewater was also used in this work. In this case, the major difference between wastewaters was the ammonium concentration, being around 1 g/L for the mainstream.

After each experiment, the remaining feed solution and, also, the ammonium salt produced were both analyzed to corroborate that only ammonium passed through the LLMC but not other elements (data not shown).

### 3.1. Effect of Changing Feed or Acid Stripping Solution between LLMC Process Steps to Increase Ammonia Recovery

**Figure 1:**
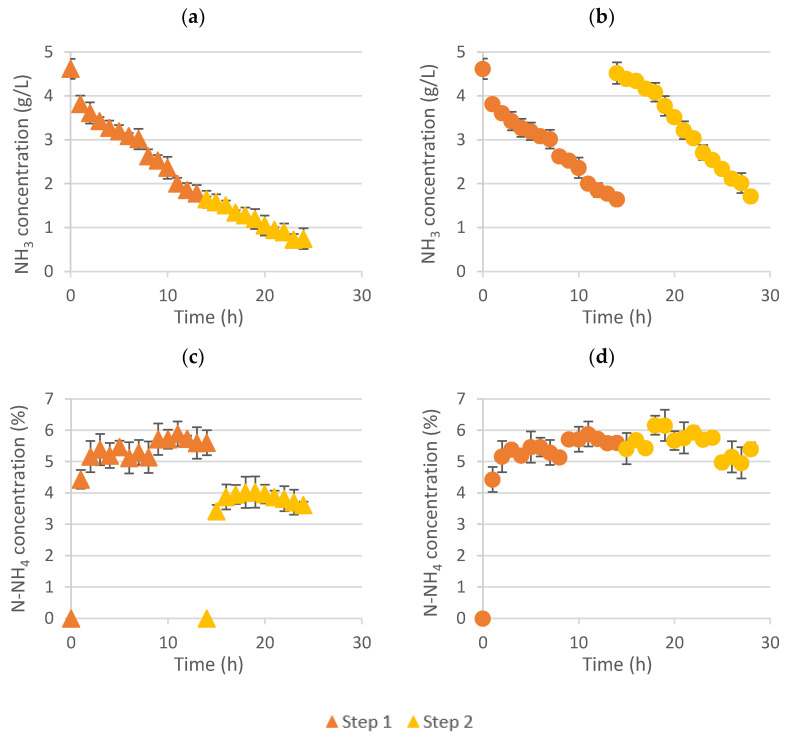
Ammonia concentration evolution over time in the feed tank when changing (**a**) the acid and (**b**) feed between steps (up). Nitrogen concentration achieved in the liquid fertilizer by changing the (**c**) acid or (**d**) feed solution between steps (down). Orange color implies one stage of LLMC (triangle for the feed side and circle referring to the fertilizer solution), while yellow color refers to experiments with two LLMC stages (triangle for the feed side and circle referring to the fertilizer solution).

As abovementioned, two scenarios were studied: (i) changing the acid solution between steps and (ii) replacing the feed solution with a new one between steps. In the first case, the idea was to further decrease the final ammonia concentration of the feed solution while obtaining two liquid fertilizer solutions of a similar concentration. In the second scenario, the aim was to achieve a more concentrated liquid fertilized in the acid stripping side, although not able to decrease the ammonia concentration of the feed solution so much. Figure 1 shows the ammonia evolution in the feed solution and the nitrogen concentration evolution in the ammonium salts solution over time for both scenarios.

As can be seen in Figure 1, the ammonia concentration in the feed solution decreased over time from around 4.5 to 1.6 g NH_3_/L during the first step (Figure 1a,b). Then, changing the fertilizer for a new acid solution (0.4 M HNO_3_) in the stripping side, it was possible to further decrease the ammonia concentration down to 0.7 g/L (Figure 1a), whereas a similar behavior than in step 1 was achieved when changing the feed solution for a new one with approximately 4.5 g NH_3_/L, reducing its concentration to 1.6 g NH_3_/L (Figure 1b).

On the other hand, comparing the nitrogen concentration evolution in the stripping side (Figure 1c,d), it can be seen that two ammonium salt solutions (around 5.4% N-NH_4_ and 3.9% N-NH_4_) were produced when changing the acid between steps (Figure 1c), whereas a unique liquid fertilizer was produced when changing the feed solution, although its concentration was almost not even increased (from around 5.5 to 5.6% N-NH_4_) (Figure 1d).

**Figure 2:**
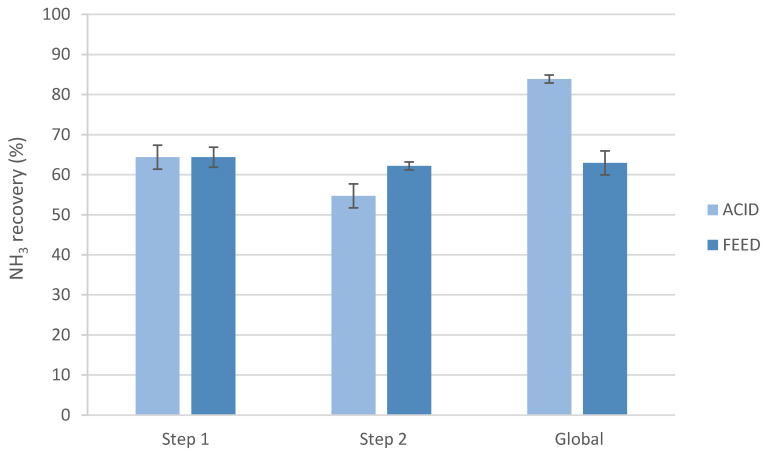
Ammonia recovery after each step, and the global results changing the acid or the feed solution between steps.

Additionally, the ammonia recovery was calculated after each step and was also determined for the global process, taking into account both steps (Figure 2).

As can be seen in Figure 2, the first step for both experiments had the same performance, achieving ammonia recovery values of around 64.4%. The results for the second step demonstrated that the NH_3_ recovery obtained was better when changing the feed solution after reaching a plateau (62.2 vs. 54.7% N-NH_4_) since the initial ammonia concentration was again the same as in the beginning, so more ammonia ions could react with the remaining nitric acid of the stripping side. Nevertheless, the global and maximum ammonia recovery achieved was higher (≈83.9%) when changing the acid stripping solution between steps.

Few papers can be found in the literature studying the effects of working with different steps of LLMCs. Indeed, preliminary experiments by two-stage LLMC changing the acid stream were previously done by our research group [10]. The results demonstrated that similar results were obtained by one or two steps. For this reason, in the present work, the feed solution was changed between steps to try to enhance the overall performance. However, changing the acid solution between steps was selected as optimal regarding the results. In fact, it allowed to obtain a feed solution with less ammonia concentration and two liquid fertilizer solutions with a similar nitrogen concentration, and also, a higher ammonia recovery (around 25% more) could be reached.

Furthermore, Zhang et al. [14] proposed a three-stage LLMC performance, changing the acid stripping solution by passing the feed stream through the three LLMC in the series. The main objective was to recover ammonia from human urine as ammonium nitrogen. The results are in agreement with the one found in this article, since the average ammonia removal percentage was much higher as a global value (over 99%), than taking into account LLMC by LLMC (between 80% and 83%). Additionally, Yan et al. [15] studied a four-stage LLMC system, recirculating both streams, feed and acid between steps. Again, the results demonstrated that the ammonia recovery could be increased by including more LLMC stages, being able to enhance the recovery value from 65 up to >98%.

### 3.2. Initial Ammonia Concentration Effect on the Overall LLMC Performance

**Figure 3:**
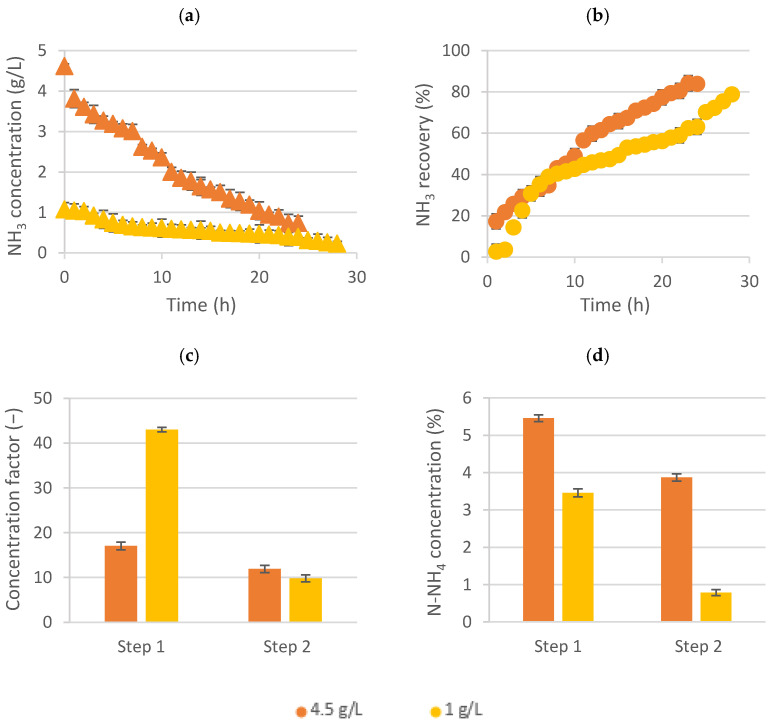
Comparison between working with the sidestream (high NH_3_ concentration) and mainstream wastewater (low NH_3_ concentration): (**a**) ammonia concentration evolution in the feed tank, (**b**) ammonia recovery, (**c**) concentration factor and (**d**) %N-NH_4_ concentration in the liquid fertilizer. High ammonia concentration is indicated by the color orange, while the color yellow implies working at low ammonia concentrations.

Sidestream wastewater (around 4.5 g NH_3_/L) and mainstream wastewater (≈1 g NH_3_/L) were used as the feed solution in the LLMC. The results indicated that both wastewaters could be treated by LLMC, although several parameters were determined to establish the optimal performance. Figure 3 shows the ammonia concentration evolution and its recovery on the feed tank (up) and, also, the concentration factor and the nitrogen concentration achieved in the ammonium salt solution (down).

As shown in Figure 3a, the final ammonia concentration was lower when working with the mainstream wastewater, being able to achieve values lower than around 200 mg NH_3_/L. In this case, this stream (ammonia-free) could be reused in the zeolites process [10]. Moreover, not only lower levels of ammonia were achieved by mainstream water but, also, a similar recovery percentage (almost 80%) in comparison when using sidestream solutions (Figure 3b).

On the other hand, since the initial ammonia concentration in the feed solution was lower, when using the mainstream (1 g/L), the concentration factor was much higher (43 times) during the first step of this experiment (Figure 3c). Nonetheless, the obtained nitrogen concentration values in the ammonium salt solution were higher when using the sidestream wastewater (around 5.5% and 3.9% N-NH_4_ in the first and second steps, respectively) (Figure 3d). Thus, if the main purpose is to obtain a more concentrated liquid fertilizer, it would be better to use sidestream wastewater. However, evaluating the feed side of the LLMC, the difference between the ammonia recovery achieved using both wastewaters was around 6%, whereas the difference between the final ammonia concentration in both cases was almost 70%. Thus, the less-concentrated wastewater had a more efficient ammonia recovery performance by LLMC, although it obtained a less-concentrated liquid fertilizer.

The initial ammonia concentration influence on LLMC experiments has previously been studied, although there is not a concluding resolution. Some authors reported that the initial concentration did not influence the ammonia mass transfer through LLMC, whereas other authors, such as in our case, concluded that a lower ammonia initial concentration allowed a better LLMC performance.

For instance, the results obtained in this work agree with the published results by Ahn et al. [16]. In that case, a PTFE 0.4-μm tubular membrane was employed with 205.5 cm^2^ of effective surface. Amongst the other parameters such as pH, feed and stripping flow rate, the influence of the initial ammonia concentration (250–1000 mg/L) was studied. The experiments were assessed in 10 h of operating time, and the results obtained were the following: when 1000 mg/L was used, the ammonia concentration decreased until reaching a concentration of approximately 300 mg/L, whilst 250 mg/L showed a lower, steep decreasing trend over time and, thus, worse removal efficiency. Nevertheless, it was possible to achieve lower concentrations at 10 h (from 250 to 100 mg/L) than working with the 1000 mg/L solution (down to 300 mg/L). Furthermore, the results indicated a lessening in the mass transfer coefficient as the ammonia concentration was increased (8.9 × 10^−3^ m/h to 7.0 × 10^−3^ m/h).

On the other hand, Ashrafizadeh and Khorasani [17] simulated different scenarios of ammonia recovery through LLMCs, assessing the parameters such as pH, initial ammonia concentration and solution velocity. Regarding the influence of the ammonia concentration on the recovery efficiency, several experiments were performed testing the feed solutions with ammonia concentrations lower than the ones tested in this study (between 50 mg/L and 800 mg/L). In that case, the authors concluded that the ammonia removal had a non-dependent behavior on the feed concentration. However, Kartohardjono et al. [18,19] also tested lower initial ammonia concentrations than the ones used in this work (from around 100–800 mg/L) to determine the influence of the initial ammonia concentration in the feed solution. The results indicated that a slightly less ammonia recovery efficiency was achieved when increasing the initial ammonia concentration. In other words, the results showed that the lower the initial ammonia concentration, the more efficient the ammonia recovery. Furthermore, the overall mass transfer coefficient decreased as the initial ammonia concentration rose.

Later, Moradihamedani [2] published a review paper reporting that neither the ammonia removal nor the ammonia mass transfer coefficient were dependent on the initial ammonia concentration of the feed stream. Contrarily, Uzkurt Kaljunen et al. [20] concluded that a higher initial nitrogen concentration had a negative impact on the mass transfer coefficient. Additionally, Yu et al. [21] recently made a comparison between the influence of the initial ammonia concentration (from 100 to 2000 mg/L) when using a conventional LLMC or an aqueous–organic membrane contactor. They postulated that the initial ammonia concentration was not influenced when using conventional LLMCs, although the best results were achieved when treating feed streams with a low ammonia concentration through an aqueous–organic membrane contactor. In fact, they concluded that lower mass transfer coefficients were reached by increasing the initial ammonia concentration.

### 3.3. Study of the Effect of Initial Feed Volume on the LLMC Trials

The acid stripping side volume during the trials was kept constant at 0.5 L. However, two feed volumes (60 and 5 L) were tested to determine the best feed/acid stripping volume ratio (120 or 10) for the LLMC performance. The results (see Table 3) indicated that a lower feed volume (5 L) resulted in a better performance on the feed side, whereas a higher feed solution volume (60 L) implied a better evolution in the fertilizer side results.

For all these experiments, mainstream wastewater was used (initial NH_3_ concentration around 1 g/L). As can be seen in Table 3, although the initial ammonia concentration was the same, more NH_3_ recovery (96.7% vs. 78.8%) and less final ammonia concentration in the feed solution (38.2 vs. 227.5 mg NH_3_/L) were achieved by working with the 5-L feed solution in comparison with the 60-L wastewater stream. Thus, it can be concluded that lees feed water permitted to remove more ammonia from the mainstream wastewater, as well as to obtain a more ammonia-free stream at a high pH, could be reused in the regeneration stage of the zeolites process. On the other hand, if the main objective is to achieve a more concentrated liquid fertilizer, better results were obtained when treating the greater volume (60 L). In this case, higher CF values (one order of magnitude higher) and more %N-NH_4_ (3.5% vs. 0.9% during the first step) were obtained in comparison to the results achieved by the lower volume experiments. Thus, more feed volume could be used if more concentrated fertilizer is required.

To the best of our knowledge, only one work was found in the literature studying the feed volume effect working with LLMCs. Indeed, the results of this work are in concordance with the results obtained by Mayor et al. [1]. In that case, three different feed volumes were tested: 5, 30 and 60 L. The results showed that the lower feed volume increased the ammonia recovery (from 85.0 to 96.3%) and also decreased the experimental time, obtaining the lower fertilizer concentration.

### 3.4. Wastewater Temperature Effect on the LLMC Process

Lastly, the effect of the temperature of the feed wastewater was studied as a variable of the LLMC performance. In this case, room temperature (25 °C) and 10 °C more (35 °C) were tested. Again, the employed feed solution was the mainstream, with an initial concentration of about 1 g NH_3_/L. Moreover, 5 L of feed solution were used.

**Figure 4:**
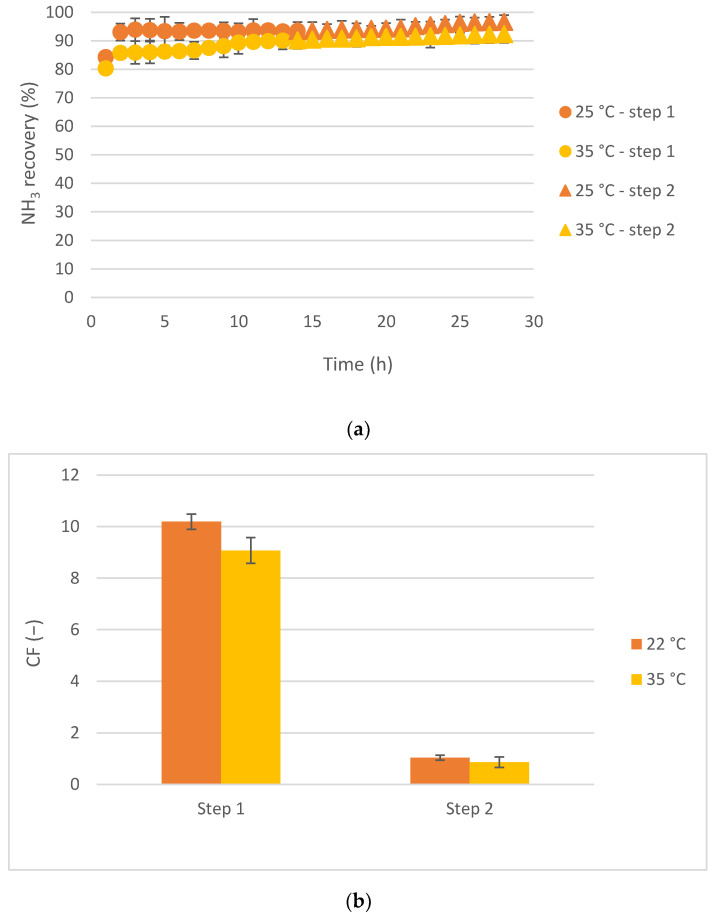
Temperature results comparison: (**a**) ammonia recovery and (**b**) concentration factor.

Figure 4 shows the ammonia recovery over time and the final concentration factor achieved working with the feed solution at 25 °C or 35 °C.

As can be seen in Figure 4a, high ammonia recovery values were obtained in both cases (about 92%). An ANOVA test was done to determine if the obtained differences were significant, obtaining a *p*-value higher than 0.05, which determined that no significant differences occurred during NH_3_ recovery when testing the LLMC with a feed solution at 25 or 35 °C. On the other hand, Figure 4b shows the concentration values achieved. As can be seen, higher concentration values (10.2 ± 0.3 and 9.1 ± 0.5 working at 25 and 35 °C, respectively) were obtained during the first step of the LLMC performance in comparison with the second step (1.0 ± 0.1 and 0.9 ± 0.2 for the 25 °C and the 35 °C experiments, respectively). Again, the concentration factors comparison between the different temperatures tested was done with an ANOVA test, obtaining a *p*-value > 0.05 for each step, indicating that the influence of the feed temperature was not significant on the overall LLMC performance.

Moradihamedani [2] recently published a review article indicating that a higher feed temperature had a positive influence on the ammonia recovery by LLMC, improving the ammonia removal. However, it was concluded that this impact was significant at temperature values higher than 40 °C. High temperatures (>40 °C) improved the ammonia partial pressure, improving the ammonia mass transfer due to the pressure gradient. However, when the temperature was lower than 40 °C, it seemed that there was no influence on the overall performance. In fact, Ahn et al. [16] tested several LLMC operation parameters, such as flow rate, stripping solution, feed wastewater pH and temperature. In that case, the temperatures tested varied by 13 °C, being 22 °C and 35 °C. The results demonstrated that, although the ammonia removal percentage slightly increased (by 4.4%) with the temperature, its effect was not significant. On the other hand, the maximum temperature allowed regarding the Design & Operational Guidelines of the LLMC Manufacturer Company (3M^TM^) was 35 °C, which did not damage the membrane contactor [22]. Regarding the results of this work and, also, the obtained results in the already published literature, it would be better to work at room temperature (22–25 °C) instead of increasing the feed wastewater temperature. Thus, similar results will be obtained, although lower operational costs will be required without heating the feed solution. If not, based on the published literature, higher temperatures than 40 °C would be recommended.

## 4. Conclusions

This work studied the effect of several operational conditions during the LLMC performance. For instance, feed or acid solution replacement between the steps was evaluated, concluding that more than 20% improvement was achieved in the ammonia removal percentage when changing the acid stripping solution. Regarding the initial ammonia concentration, considering a sidestream (4.5 g NH_3_/L) or a mainstream (1 g NH_3_/L), both streams could be used with good LLMC results, depending on their purpose. A sidestream could be useful when the maximum ammonia recovery (6% difference) and more %N-NH_4_ is required, whereas the mainstream would be better able to decrease the feed ammonia concentration to a lower value and, at the same time, to reach a higher concentration factor (60% of the difference in the first step). Additionally, the feed volume also has an impact on the LLMC technique. In fact, a higher volume has a more positive impact on the fertilizer side (more concentration factor and %N-NH_4_), while a lower volume implied better results in the feed side (more ammonia recovery and less final ammonia concentration in the feed side). Finally, the differences in temperature tested were not enough to have significant improvements in the LLMC performance. All in all, LLMC proved to be a versatile technique to treat wastewater with low (1 g/L) and high (4.5 g/L) initial ammonia concentrations, where the most influential parameter was the change of the stripping solution in the formulation of ammonium salts. Therefore, LLMC could be an easy technique to be implemented in biofactories for the recovery of nutrients from main or side wastewater streams.

## Figures and Tables

**Table 1:** Experimental design.

	Exp. 1	Exp. 2	Exp. 3	Exp. 4	Exp. 5
Change between steps	acid	feed	acid	acid	acid
NH_3_ concentration (g/L)	4.5	4.5	1	1	1
Volume (L)	60	60	60	5	5
Temperature (°C)	25	25	25	25	35

**Table 2:** Initial sidestream wastewater composition.

Parameter	Value	Units
Sodium (Na^+^)	12.70 ± 0.01	mg/L
Ammonium (NH_4_^+^)	4.60 ± 0.14	
Potassium (K^+^)	0.46 ± 0.05	
Magnesium (Mg^2+^)	0.03 ± 0.01	
Calcium (Ca^2+^)	0.04 ± 0.02	
Chloride (Cl^−^)	0.35 ± 0.14	
Nitrate (NO_3_^−^)	0.33 ± 0.11	
Phosphate (PO_4_^3−^)	0.05 ± 0.02	
Sulphate (SO_4_^2−^)	0.38 ± 0.11	
pH	13.13 ± 0.24	-
Conductivity	66.30 ± 0.99	mS/cm
Total carbon (C)	57.93 ± 0.87	mg/L

**Table 3:** Results of the study of the feed volume effect.

		60 L			5 L		
		Step 1	Step 2	Global	Step 1	Step 2	Global
Feed side	NH_3_ recovery (%)	47.5 ± 0.9	59.7 ± 1.1	78.8 ± 1.8	93.5 ± 3.2	48.6 ± 0.8	96.7 ± 2.9
	Final [NH_3_] (mg/L)	564 ± 55.6	227.5 ± 49.9		74.4 ± 8.2	38.2 ± 5.3	
Acid stripping side	CF (−)	43.0 ± 0.8	9.8 ± 0.8	-	10.2 ± 0.6	43.0 ± 0.8	9.8 ± 0.8
	%N-NH_4_ (%)	3.5 ± 0.1	0.8 ± 0.1	-	0.9 ± 0.1	3.5 ± 0.1	0.8 ± 0.1

## Data Availability

Not applicable.
